# Restoring to Its Natural Appearance a Putrified Dead Body

**Published:** 1863-07

**Authors:** Benjamin Ward Richardson

**Affiliations:** Senior Physician to the Royal Infirmary for Diseases of the Chest


An Account of an Attempt to Restore to its Natural Appearance a Putrified Dead Body, in order to Prove its Identity.—*By Benjamin Ward Richardson, M.A., M.D., Senior Physician to the Royal Infirmary for Diseases of the Chest.*—On Saturday, the 9th instant, I conducted an inquiry to ascertain if a human body that had undergone putrefactive change to such a degree that it was unrecognizable, could be so far restored to the appearance of life as to be sworn upon in respect to its identity.

As the inquiry in question, from the circumstances by which it was surrounded, has created great public interest, as it opens a new line of research in regard to a medico-legal question of a very important nature, and as certain imperfect impressions are afloat concerning it, I take the opportunity of laying the exact scientific facts before the profession at the earliest possible moment.

To make every point clear to provincial and foreign brethren, let me state the simple narrative of the facts in the first place. Some weeks ago a woman named Emma Jackson was murdered in St. Giles's by having her throat cut in a house of ill-fame, to which she had retired with a man who had been seen by at least three persons, and whose appearance was clearly defined by them. This man, by some strange and almost inexplicable method, made his escape from the house without being seen to depart, and has not since been detected. Several persons have, however, been suspected and one or two have been temporarily detained, but on examination they have been discharged.

On Monday, May 4th, a man was dragged dead from the Thames, who, in many respects, seemed to answer to the description given of the assumed murderer. On the following Wednesday, Mr. Humphreys, the coroner for East Middlesex, held an inquest on the body of this man, but decomposition had advanced so far that none of the witnesses could arrive at any conclusion whatever respecting the body; it was, in fact, utterly unrecognizable. This statement having been made in the public papers on Thursday morning, I formed an opinion, derived from some researches on dead tissues, that it might be possible to alter the appearance of the body so much as to enable the witnesses to speak as to its identity. In the afternoon of Thursday last I met, accidentally, Dr. Lankester, who had held the inquest over the body of Emma Jackson, and I explained to him my views. He urged me very strongly to communicate with Mr. Humphreys. I did so, and through the kind aid of Dr. Edmunds got an interview with Mr. Humphreys on Friday night. Having given him an outline of the plan I proposed to follow, he deputed me to carry out the attempt, and requested Dr. Edmunds to be present and take part in conducting the suggested process. We were to act at once, as the adjourned inquest was to be held on Saturday.

At half past ten on Saturday we were taken to the dead man, who was lying in a shell in the dead-house on Darby street, Tower Hill. He was dressed as he was when taken out of the water. His body generally, with the exception of the hands, was deeply discolored, and the face was so changed that it was quite impossible to form any opinion respecting either its color or feature; it was as black as the face of the darkest negro, and had it not been white when he was taken out of the water, I should say that the man would have been returned as a negro. The lips were enormously distended, and the nose was scarcely visible; the cheeks and eyelids were also greatly distended. In fact, the putrefactive changes were so advanced that it required some little determination to proceed. Following, nevertheless, the course I had marked out, we immersed the body in water, and then added to the water twenty pounds common salt; we also added gradually, in the course of the operation, one pint of common hydrochloric acid; and the body was allowed to remain under this solution for two hours. The object of this part of the process was to reduce the swelling of the features by exomosis. The shell, being water tight, answered as a bath.

Meanwhile we charged a pail of water with fresh chlorine, and then, lifting the face out of the water in the shell, treated it with the chlorine water. I also directed a stream of chlorine gas for some time upon the face. The object of this part of the process was to restore the white color.

A little before one o'clock, both of the intentions we had in view were realized to a considerable degree. The tumefaction was relieved; and the face, from the deepest black, had become of the cast of light clay, common wood-ash, or the darker sort of straw paper. When the chlorine in vapor was passing over the face, the skin approached to white, but so soon as it was withdrawn the change to clay-like hue returned. So much was now accomplished that we were able to form a fair estimate of the man. We found that he was evidently a young man, not more probably than twenty-one years of age; he had a short, feeble moustache; his lower lip had a short, soft beard that had not been shaven, and his whiskers corresponded; his face was naturally round and full, and his body generally was well nourished.

At one o'clock we left, and returned at two. We had arranged that a stream of chlorine should continue to play over the face in our absence, but, as we had no one to leave in charge, the gas became exhausted, and the face was a little darker when we returned.

Pursuing still the course I had prearranged, we opened the body. We found the viscera but little decomposed and natural; the heart was empty and flaccid; the lungs free from congestion. We fixed a large tube in the aorta, through the left ventricle; and Dr. Edmunds tied the aorta in the thorax, so as to prevent any passage of fluid to the lower part of the body, and to the abdominal viscera. Then we injected a solution, consisting of chlorine water, chloride of zinc, and a little sesquichloride of iron. The object in this instance was to impregnate the tissues from within with the decolorizing agent, and to reduce the tumefaction. On forcing the injection, we found that great escape took place through the vessels that had been divided in opening the thorax. We therefore withdrew the tube from the aorta, and as the face was the part chiefly requiring attention, Dr. Edmunds laid bare the common carotid on the right side, and a small nozzle from the syringe was introduced into that vessel and tied. It must be understood that much care was required in forcing the injection through structures so decomposed and yielding, and that we dare not push this part of the operation too far. Had we used much force we should have produced extensive infiltration through the broken capillaries, and have destroyed the facial structures altogether. So soon, therefore, as the face was subjected to slight tension, the injection process was stopped. The time had now approached for the sitting of the jury at half past 4 P. M. We allowed all the water to drain away, drenched the body with pure water, and left it with the face covered with a piece of thick cloth, on which was poured a little hydrochloric acid and methylated alcohol. The face at this time was of a clayey color, and a little more full than natural; and although we felt that we had not brought it up to its perfect natural appearance, we believed that it might be recognizable by any one who had seen it during life, and especially that it was a face which a witness could swear was not that of any particular person whom he remembered, if there were not strong natural resemblances between the two.

The result indicated that we had effected even more than we had anticipated, and that, if we had not succeeded to the perfection we could have wished, we had fulfilled the practical part of our mission, and all that was demanded of us; for the three witnesses who were there either to confirm or disprove the hypothesis that the man before them was the man last seen with the murdered woman, each and all swore without hesitation, on their second view of the unknown man, that he was *not* the assumed murderer.

Margaret Curley, of 4 George street, St. Giles's, swore that she had examined the deceased since the operation had been performed, but that she did not recognize him as a per son she had seen before, nor as the person suspected; Charles Ansley, of 20 Peter street, bore the same testimony; and H. Stoke, the shoe-man, swore definitely that, from his inspection of the deceased since the operation, he was sure that he was not the man whom he had seen with Emma Jackson. The Coroner, in summing up, observed that the experiments made having enabled the witness to swear that the deceased man was not the man accused of the murder, they had fulfilled their purpose, and the jury returned a verdict in accordance with the evidence.

*Reflections and Suggestions.*—The fact that in a case so extreme as the one named, science has come in to render essential aid to justice, affords, I hope, subject for thought and renewed effort in the same direction. I am far from considering that we ought to stop where we have thus begun. I look upon this case, in fact, as a mere first and experimental trial, which, followed up, will lead to great perfection in one department of medical jurisprudence; and I feel, consequently, that I can not conclude this paper better than by pointing out what improvements in the process have been suggested to me by the experience detailed above.

1. In respect to time. On another occasion I would ask to be allowed at least twenty-four hours for the performance of the process. The period of six hours was insufficient for the full development of the required changes.

2. I should proceed by stripping the subject of all apparel.

3. After this the subject should be placed in a water-tight shell, in which a large tap for escape of water should be inserted, and the body should be thoroughly washed with water.

4. After the washing, the body should be covered with water, and held beneath it by a few cross bars of wood. Then the lid of the shell should be temporarily but effectually closed down, and two openings should be made into the lid; through one of these openings the free end of a tube, connected with a chlorine flask, should be passed beneath the surface of the water; while from the other opening should come another tube, the free end of which should turn over into a glass globe of water. These preliminaries arranged, fresh chlorine should be driven in until the water within is saturated by it, the fact of saturation being determined by the passage of chlorine through the escape-tube. When the water around the body should thus become charged with chlorine, the openings in the lid of the shell should be closed, and the whole should be left undisturbed for twelve hours.

5. On opening the lid after the interval of time named, common salt should be added to the water, until the hydrometer should stand several degrees above the specific gravity of the blood; the specific gravity of 1100 would answer for the solution. In this solution the body should remain immersed for twelve hours; the water should then be drawn off and the body examined.

[If there were no deep decomposition and discoloration, the body, I believe, would now be ready for identification; but if the putrefaction were very deep-seated, it would be requisite to proceed further.]

6. If necessary, open the trunk of the body at this point, and make any post-mortem examinations that may be required. The head should not be opened at this stage.

7. After the post-mortem examination, in order to restore a more natural expression to the face, solutions should be injected into the external carotid of each side. The form of solutions I should suggest in another case would be—

(*a*) Water saturated with chlorine, and charged, in addition, with tincture of the sesquichloride of iron in the proportion of two fluid drachms to the pint.

(*b*) Common fresh milk saturated with common salt.

Of injection *a*, I would recommend that from two to three ounces should be slowly injected on each side, to be followed, without removing the nozzle of the syringe from the vessel, by so much of solution *b* as should cause the slightest possible tension on the tissues of the face.

Lastly, if it were necessary to retain the body for some time, it would be advisable to cover it with wood spirit, containing one drachm to the gallon of the tincture of the sesquichloride of iron, and to exclude it from the air.

In offering these suggestions, I beg that they may be accepted as open to revision; the principle recognized, the details are certain, under experiment, to be simplified and improved.

In conclusion, I have to offer my warmest thanks to Dr. Edmunds, for the energetic, friendly, and able part which he took in the very interesting inquiry to which I have called attention. His exertions contributed in a most important manner to the results obtained.—*London Lancet*, May 16, 1863.

